# Biomusic: An Auditory Interface for Detecting Physiological Indicators of Anxiety in Children

**DOI:** 10.3389/fnins.2016.00401

**Published:** 2016-08-30

**Authors:** Stephanie Cheung, Elizabeth Han, Azadeh Kushki, Evdokia Anagnostou, Elaine Biddiss

**Affiliations:** ^1^Institute of Biomaterials and Biomedical Engineering, University of TorontoToronto, ON, Canada; ^2^Bloorview Research Institute, Holland Bloorview Kids Rehabilitation HospitalToronto, ON, Canada; ^3^Department of Paediatrics, University of TorontoToronto, ON, Canada

**Keywords:** sonification, disability, anxiety, augmentative and alternative communication (AAC), music

## Abstract

For children with profound disabilities affecting communication, it can be extremely challenging to identify salient emotions such as anxiety. If left unmanaged, anxiety can lead to hypertension, cardiovascular disease, and other psychological diagnoses. Physiological signals of the autonomic nervous system are indicative of anxiety, but can be difficult to interpret for non-specialist caregivers. This paper evaluates an auditory interface for intuitive detection of anxiety from physiological signals. The interface, called “Biomusic,” maps physiological signals to music (i.e., electrodermal activity to melody; skin temperature to musical key; heart rate to drum beat; respiration to a “whooshing” embellishment resembling the sound of an exhalation). The Biomusic interface was tested in two experiments. Biomusic samples were generated from physiological recordings of typically developing children (*n* = 10) and children with autism spectrum disorders (*n* = 5) during relaxing and anxiety-provoking conditions. Adult participants (*n* = 16) were then asked to identify “anxious” or “relaxed” states by listening to the samples. In a classification task with 30 Biomusic samples (1 relaxed state, 1 anxious state per child), classification accuracy, sensitivity, and specificity were 80.8% [standard error (SE) = 2.3], 84.9% (SE = 3.0), and 76.8% (SE = 3.9), respectively. Participants were able to form an early and accurate impression of the anxiety state within 12.1 (SE = 0.7) seconds of hearing the Biomusic with very little training (i.e., < 10 min) and no contextual information. Biomusic holds promise for monitoring, communication, and biofeedback systems for anxiety management.

## 1. Introduction

Medical advancements have led to a growing number of people surviving previously fatal medical complications, and subsequently living with profound disabilities. For these individuals, survival depends on life-supporting technologies and teams of caregivers who can anticipate and respond to their complex continuing care needs. Even when cognitive function remains intact, many of these individuals are unable to communicate via traditional pathways (e.g., speech, gestures) and/or augmented and assisted communication (AAC) devices (Hogg et al., 2001) because of physical limitations. As such, it often falls to caregivers to decipher their preferences, intent, and emotions from sparse behavioral and contextual cues (Adams and Oliver, 2011; Blain-Moraes et al., 2013) that may be difficult to detect, non-obvious to those unfamiliar with the individual, and inconsistent/unavailable across individuals (Adams and Oliver, 2011). In the absence of a reliable communication pathway, the needs, thoughts, and feelings of those with profound disabilities are at risk of being overlooked, which presents concerning challenges to health and well-being (Blain-Moraes et al., 2013). This motivates the urgent need to establish communication channels with individuals with profound disabilities and methods for discerning their emotional states.

Emotion-modulated physiological signals may provide additional cues when interpreting the affective states of those with profound disabilities. Vos et al. (2012) have demonstrated that changes in skin temperature and heart rate may be associated with positive and negative valence emotions in persons with profound disabilities. While several groups have developed algorithmic classifiers that use machine learning to identify and display affect from physiological signals of healthy adults (Wen et al., 2014), the application of this research to individuals with profound disabilities presents an additional difficulty. Methodologically, it is extremely challenging to develop classifiers with a population who are unable to verify their performance or communicate the “ground truth.” Ethically, we must be conscious of the potential challenges of assigning affective state labels to individuals who can neither confirm nor correct their accuracy.

An alternative is to consider physiological signals as an additional source of information that can be continuously streamed, rather than discretely classified. Caregivers may learn to interpret this information and integrate it alongside other contextual and behavioral cues. In contrast to a computerized classifier, this approach relies on human intelligence and aptitude for pattern recognition. It does not presume to assign labels, but rather hinges on the ability to effectively present multiple complex, streaming physiological signals to caregivers in a way that is easy and intuitive to understand. Reliance on human interpretation may introduce variability, but it may also offer more flexibility to accommodate a wider range of person- and condition-specific heterogeneity, to base decisions on multiple information sources (e.g., physiology, behavior, context), and to avoid ethical challenges associated with assigning definitive emotion labels.

Even for experts, it may be extremely challenging to make sense of complex raw physiological data (Sanderson, 2006). Intuitive auditory displays could make the valuable information contained in these signals more accessible. To this purpose, we created Biomusic, an auditory interface that converts physiological signals to sound. The system outputs sounds in MIDI format, which can be listened to in real-time or offline. Specifically, the music's melody is driven by changes in electrodermal activity; the musical key, established by tonic chords, is linked to changes in skin temperature; a rhythmic, percussive beat is associated with heart rate (extracted from blood volume pulse); and respiration is projected via a “whooshing” embellishment resembling the sound of an exhalation (Table 1). Mappings were based upon guidelines from Watson and Sanderson, who suggested that periodic signals such as heart rate may be naturally suited to tempo, while aperiodic signals may suit tonal representation (Watson and Sanderson, 2001). Thus, as per criteria outlined by Hermann (2008), Biomusic is objective (i.e., reflective of the nature of the inputs) and systematic in its mapping, is able to reproduce its output given the same input, and can be applied to physiological signals of multiple different users.

**Table 1:** **Physiological Signal-to-Music Mapping**.

**Physiological signal**	**Feature extracted**	**Musical representation**
Electrodermal activity	Average skin conductance over a 0.25-s window in μS	Melodic pitch (“goblins” MIDI-sound): Three octaves of a major-key scale are mapped to 1 μS of electrodermal activity. The melody establishes the first windowed and averaged electrodermal activity value as the tonic center in a major scale. Subsequent EDA values are compared to the first, and differences result in a pitch shift relative to the magnitude of change. Extreme levels of electrodermal activity are transposed to keep the melody in a comfortable range. Thus, quickly-changing EDA results in active melodic runs. Melody may be suited to reflect the salience of continuous changes in electrodermal conductivity with anxiety state.
Skin temperature	Instantaneous skin temperature in degrees C	Key change (“choir” MIDI-sound chords): Major tonic chords are mapped to instantaneous skin temperature. This feature is updated every two bars. The first skin temperature value is mapped to the tonic chord of a C-major scale, and subsequent increases or decreases in temperature raise or lower the key of the music, and therefore the chord, by one semitone. Chords indicative of key change may be suited to represent gradually-continuous changes in skin temperature.
Blood volume pulse	Average interbeat interval over four periods of the signal	Tempo (“melodic tom” MIDI-sound): The pace of the music, marked by a percussive beat, is updated every musical bar. This tempo is determined from the average interbeat interval of the blood volume pulse signal over four periods. Therefore, increased heart rate increases the tempo of the music and its beat. A drum beat is suited to represent the rhythmic, cyclic pulse of the circulatory system.
Respiration	Duration of exhalation (peak-to-trough intervals of the signal)	“Whoosh”: Expiratory time is mapped to the duration of a whooshing, seashore embellishment resembling the sound of an exhalation. The “Whoosh embellishment is sustained as long as exhalation is detected. During inhalation, this sound is not played.

While visually-streamed physiological data demand continuous attention to interpret, Biomusic can provide caregivers with peripheral awareness of multiple physiological signals and trends while preoccupied with other aspects of care (Blain-Moraes et al., 2013). In a systematic review, Dubus and Bresin (2013) identified and categorized 7 sonification projects as having the primary purpose of “monitoring.” This suggests the utility of an auditory medium such as Biomusic to convey continuous, real-time information to an attentive listener. Previous studies have demonstrated the effectiveness of using sound to represent physiological signals. Yokoyama et al. (2002) mapped instantaneous heart rate to musical pitch and intervals for use in biofeedback for stress and blood pressure management. Wu et al. (2009) enabled participants to distinguish between rapid-eye movement and slow-wave sleep when presented with sonified electroencephalographs. As such, there is evidence to suggest that complex physiological signals can be interpreted by listening to their musical translations. Unlike these interfaces, Biomusic enables information from multiple physiological signals to be projected simultaneously through different but concurrent mappings, forming a holistic soundscape. To our knowledge, Biomusic is unique in this regard.

Biomusic has been successfully demonstrated in a clinical care context to augment communication between caregivers and children with profound disabilities by increasing a sense of reciprocity and co-presence, two fundamental qualities of human interaction (Blain-Moraes et al., 2013). Anecdotally, caregivers in this study noticed changes in the sound and quality of Biomusic that appeared to be associated with their interactions with the children. Caregivers in that study expressed a strong desire to decode or interpret Biomusic, which motivated the research question reported on in this paper: How effectively can affective states, specifically anxiety, be identified using Biomusic? The present study aimed to formally evaluate how well different physiological patterns associated with affective state can be detected via Biomusic. Specifically, we determined the accuracy and speed with which listeners with minimal training could distinguish anxious from relaxed physiological responses based on Biomusic alone. Anxiety was selected as the target emotion given its clinical importance and its suspected prevalence in individuals with profound disabilities. In typical populations, anxiety modulates multiple physiological signals through excitation of the sympathetic nervous system and inhibition of the parasympathetic nervous system (Kreibig, 2010). This results in:

- Increased electrodermal activity due to increased perspiration (Vetrugno et al., 2003).
- Decreased fingertip skin temperature due to vasoconstriction (Rimm-Kaufman and Kagan, 1996).
- Increased heart rate due to increased cardiac contraction (Franchini and Cowley, 2004).
- Increased respiration rate resulting from faster, shallower breathing (Kreibig, 2010).

Through two experiments, we aimed to determine the feasibility of the Biomusic interface for conveying anxiety-related changes in physiological responses. Two experiments were conducted and in each, the following approach was used. First, physiological recordings were collected from children during relaxed and anxiety-provoking conditions. Next, anxious and relaxed state Biomusic samples were generated from these physiological recordings. Last, adult participants were asked to listen to the Biomusic samples and to identify the anxiety state associated with each, based on the Biomusic alone and no additional contextual cues. Anxious and relaxed state Biomusic samples are included in Supplementary Materials.

In the first experiment, we generated Biomusic samples from children who were considered to be typically-developing and represented the best case use scenario, having typical physiological signals and the ability to report emotions to corroborate their anxiety state. The second experiment followed a protocol identical to the first, but with the addition of Biomusic samples generated from children with autism spectrum disorders (ASD). Children with ASD have been reported to have atypical anxiety-modulated physiological responses and difficulty recognizing and expressing emotional state (Kushki et al., 2013). The ASD group represents a stepping-stone population as we work toward the eventual use case: a child with profound disabilities, with possibly typical or atypical signals, and no ability to report emotion.

## 2. Experiment 1: detection of anxiety state through biomusic in typically developing children

In the first experiment, we tested the feasibility of Biomusic technology to convey anxiety by having adult raters classify samples of Biomusic. We chose to use the Biomusic of typically-developing children, who represent the best case use scenario, having typical physiological signals and the ability to report emotions to corroborate their anxiety state. In this experiment, we aimed to (1) quantify the performance (sensitivity, specificity, accuracy) of physiological sonification as an anxiety screening tool, (2) determine the latency in seconds for detecting an anxious or relaxed state, (3) explore the confidence with which detection are made, and (4) identify the most influential musical elements in the decision. All protocols were reviewed and approved by the science and ethical review board at Holland Bloorview Kids Rehabilitation Hospital. Informed consent and assent were obtained from adults and children respectively.

### 2.1. Methods

#### 2.1.1. Biomusic

Biomusic samples were created from physiological signals of typically-developing children (aged 8–13) acquired during anxiety-inciting and relaxing conditions. Signals were acquired in individual sessions. Physiological recordings were obtained using the ProComp Infiniti data acquisition system and Biocomp Infiniti software (Thought Technology, Montreal, Canada). Four physiological signals were collected using non-invasive sensors on the fingers of the non-dominant hand (electrodermal activity, skin temperature, blood volume pulse) and chest (respiration). Electrodermal activity was recorded using dry Ag/Ag-Cl sensors, which were placed on intermediate phalanges on the second and third fingers of the non-dominant hand. Skin temperature was measured by a surface thermistor, fixed to the distal phalanx of the fifth finger, and blood volume pulse was monitored by an infrared photoplethysmography sensor, attached to the distal phalanx of the fourth finger. Respiration was recorded using a piezoelectric belt positioned around the thoracic cage. Children were instructed to limit unnecessary movements to avoid interfering with the sensors. All physiological signals were sampled at 256 Hz and passed through a 5th order Butterworth anti-aliasing filter built in to the Biocomp Infiniti.

**Figure 1:**
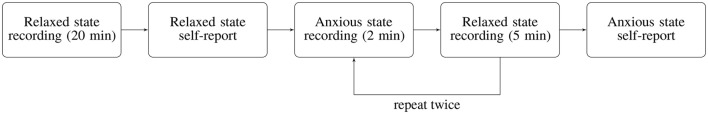
**Physiological signal acquisition procedure in Experiment 1**.

Data acquisition began with a 20-min relaxed state condition while children watched a low-intensity nature video. Jennings et al. (1992) have suggested that tasks that require some attention may be more representative of physiological “baseline” states than those that do not. Following this period, children self-reported their anxiety state using the gold standard, the child version of Spielberger's State Trait Anxiety Inventory (STAI) (Spielberger et al., 1970). The STAI is composed of two sub-scales to measure state (i.e., situational) and trait anxiety. In this study, we used the state anxiety scale. The lowest and highest possible state scores on the raw scale are 20 points and 60 points, respectively. These scores are then normalized according to Table 2 in the STAI manual (Spielberger et al., 1970). Anxiety state recordings were then collected in three 2-min trials which required children to solve anagrams of varying difficulty levels. Test-taking situations are a common method for provoking anxiety in children and have been shown to increase state anxiety on the STAI (Spielberger et al., 1970). Anagram tests have previously been used with children 8–12 years of age (Kaslow et al., 1983; Ward et al., 1987; Affrunti and Woodruff-Borden, 2014). Each anxious-state trial was followed immediately by a 5-min break during which the child engaged in the relaxed-state condition (watching a nature video). Following the anxious-state trials, children reported state anxiety on the STAI, focusing on how they felt during the anagram tasks. This protocol is outlined in Figure 1. **Table 4** presents the means of the physiological signal features of interest during the relaxed and anxiety-provoking conditions.

**Table 2:** **Means and standard error of physiological features recorded in Experiment 1 from typically developing children (*n* = 5) during the relaxed state condition and the anxiety-provoking condition**.

**Typically-developing (anagram)**		
**Signal feature**	**Relaxed mean**	**Anxious mean**
EDA (μS) ^*^	1.131, SE = 0.390	3.550, SE = 0.706
Skin temperature (°C)	32.086, SE = 1.826	30.412, SE = 2.312
Blood volume inter-pulse interval (s)	0.797, SE = 0.049	0.761, SE = 0.025
Respiration inter-breath interval (s) ^*^	3.333, SE = 0.296	2.269, SE = 0.107

* *Indicates features that were significantly different (p < 0.05) between relaxed and anxious state conditions*.

The physiological data were considered valid only if children's self-reported anxiety state matched the experimental condition (e.g., the child reported higher state anxiety while doing the anagram task). This required an increase in state score between relaxed and anxious conditions (Spielberger et al., 1970). Not all child participants may have experienced anxiety; several reported that they liked the anagram task and found it enjoyable. Of 12 child participants, 7 children [mean = 10 years, standard deviation (SD) = 2 years] met the validity criteria outlined. Of these participants, two had fidgeted excessively during data collection. Data from these two participants were not used. In the remaining five participants, the average difference in normalized state anxiety scores between relaxed and anxious states was 6.2 points (SE = 1.7) (Range: 31–52 for relaxed and 42–57 for anxious). By referring to children's self report to verify that the experimental condition had been met, we established, as well as possible, the emotional state of the children.

Once the emotional state of the physiological data was verified through referral to self-report, one anxious state and one relaxed state data segment (80-s in length) were randomly extracted from each child's physiological recordings. To avoid selecting transitional states (i.e., relaxed-to-anxious or anxious-to-relaxed), segments at the beginning and end of experimental conditions were not used. This resulted in 10 segments of physiological data (5 anxious-state, 5 relaxed-state) that were translated into clips of Biomusic as per Table 1 and recorded to be used offline. Of note, physiological signals were not referred to in the generation of the Biomusic excerpts so as not to bias the selection of songs.

#### 2.1.2. Participants

Sixteen university student participants (11 female, 5 male, 21.1 ± 2.6 years) were recruited through community postings to classify the 10 segments of Biomusic as either anxious or relaxed state. All participants had normal or corrected to normal hearing. This sample was highly representative of the volunteer population at Holland Bloorview Kids Rehabilitation Hospital—a number of whom interact routinely with children with profound communication disabilities in therapeutic recreation programs, school programs, and in their day-to-day care.

#### 2.1.3. Evaluation protocol

The feasibility of the Biomusic interface was evaluated for sensitivity, specificity, accuracy, and latency as follows. The classification session began with a brief 10-min training period during which one example of relaxed-state Biomusic and one example of anxious-state Biomusic were played. The experimenter explained the sounds that might be expected based on current understanding of the psychophysiology of anxiety (e.g., accelerated drum beat, shorter and faster whooshes, larger and more frequent runs, and changes in the melody). These 80-s training samples were generated using sections of a typically-developing child's signal recordings, which exhibited the expected anxiety-modulated physiological trends. The training samples were not included in the test set.

**Figure 2:**
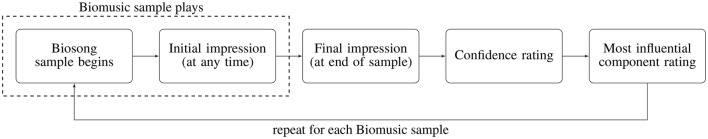
**Signal classification procedure**.

Following the training period, participants were asked to listen to all 10 test samples of Biomusic, presented in random order, and to classify each one as either “anxious” or “relaxed” state. Samples were randomized with respect to the child and to the anxiety condition. Participants had access only to the Biomusic and had no information regarding the child or the context during which the physiological signals were recorded. Participants listened to the songs in a quiet room with only the experimenter present. The songs were presented via a conventional set of computer speakers. Participants were instructed to classify the anxiety state of each Biomusic sample as soon as they felt confident in their decision. This first classification is referred to throughout this paper as their “initial impression.” The selection (i.e., anxious or relaxed state) was made on a computer screen and the time of response was recorded. After the Biomusic sample was heard in its entirety, participants indicated their final classification decision. Participants then rated their confidence in their final decision on a 5-point Likert scale ranging from 1 (low) to 5 (high). Participants also reported the most influential musical component (i.e., melody, chords, drum beat, or “whoosh”) on their classification. This protocol is described in Figure 2.

#### 2.1.4. Data analysis

The primary analysis was to determine the sensitivity, specificity, and overall classification accuracy of the Biomusic interface for classifying relaxed and anxious states from physiological signals of typically-developing children. Additionally, the latency (i.e., the average length of time required for the participants to form an initial impression of the Biomusic sample) was recorded. The median self-reported confidence was calculated to evaluate listeners' certainty during the classification task. Last, frequency counts were compiled to determine the most influential musical component in the interpretation of the Biomusic.

### 2.2. Results and discussion

On average, participants made initial classifications of the Biomusic with high accuracy [83.9%, standard error (SE) = 2.9%], sensitivity (87.1%, SE = 3.4%), and specificity (80.6%, SE = 3.4%) (Table 3). This indicates that participants could accurately classify relaxed or anxious states in typically-developing children with very little (i.e., < 10 min) training and no contextual information. On average, participants listened to the Biomusic for 12.1 [standard error (SE) = 0.7 s] seconds before registering their initial impression of its associated emotional state. Similarly, classification accuracies for the final impression (registered after listening to the full 80 s sample) were comparable: accuracy (83.8%, SE = 2.4%), sensitivity (90.0%, SE = 3.2%), and specificity (77.5%, SE = 3.6%) were high. This suggests that 12 s of the Biomusic is enough to form an accurate impression of its associated emotional state.

**Table 3:** **Biomusic performance (initial classification)**.

**Experiment 1 Biomusic**	**Accuracy (%)**	**Sensitivity (%)**	**Specificity (%)**
Typically-developing (anagram)	84.9, SE = 2.9	89.7, SE = 4.1	80.0, SE = 4.5
**Experiment 2 Biomusic**	**Accuracy (%)**	**Sensitivity (%)**	**Specificity (%)**
Typically-developing (Stroop)	82.4, SE = 3.9	91.7, SE = 3.9	73.3, SE = 5.1
ASD	83.4, SE = 3.9	83.2, SE = 6.5	83.6, SE = 3.6
Typically-developing (anagram)	84.8, SE = 3.8	85.0, SE = 4.4	85.0, SE = 4.4
All (Experiment 2)	83.9, SE = 2.9	87.1, SE = 3.4	80.6, SE = 3.4

Participants reported high confidence in their ability to classify emotional states based upon the auditory information presented through Biomusic. For both anxious and relaxed Biomusic, the median confidence rating was 4 [interquartile range (IQR) = 2] on a 5-point Likert scale ranging from 1 (low) to 5 (high). Melody was reported by participants as the most influential component for 56.9% of the Biomusic excerpts, followed by drum (25.0%), chords associated with key changes (9.4%), and the cyclic whoosh (8.8%). This trend was apparent for both Relaxed and Anxious state songs.

This first experiment demonstrates the feasibility for Biomusic to convey the information necessary for a listener to distinguish anxious from relaxed state in typical physiological signals.

## 3. Experiment 2: detection of anxiety state through biomusic in typically developing children and children with autism spectrum disorders

Having demonstrated that Biomusic could allow discrimination between relaxed and anxious states of typically-developing children to quickly, with high performance, and high classification confidence, we sought to determine if these measures would be similar in Biomusic recorded from children with autism spectrum disorders (ASD). In this experiment, adult participants classified the Biomusic of both typically developing children and children with ASD. In comparison to typically-developing children, children with ASD may have atypical physiological responses during anxiety (Kushki et al., 2013). Children with ASD have been found to exhibit raised heart rate during non-anxious and anxiety-inciting situations, a narrower range of electrodermal activity differences between non-anxious and anxiety-inciting conditions, and atypically anxiety-modulated skin temperature (Kushki et al., 2013). This population may also have difficulty recognizing and expressing their feelings (Lang et al., 2010; Hallett et al., 2013). These children represent a stepping-stone population as we work toward the eventual use case: a child with profound disabilities, with possibly typical or atypical signals, and no ability to report emotion. All protocols were reviewed and approved by the science and ethical review board at Holland Bloorview Kids Rehabilitation Hospital. Informed consent and assent were obtained from adults and children respectively.

### 3.1. Materials and methods

#### 3.1.1. Biomusic

We generated 20 Biomusic samples from a new set of children whose signals were not used to generate Biomusic in Experiment 1. These children had participated in a separate research study at our institution, through which physiological signals had been recorded in anxious and relaxed conditions. 10 Biomusic samples were created from physiological signals of typically-developing children (mean = 10 years, SD = 2 years), and 10 Biomusic samples were created from physiological signals of children with ASD (mean = 11 years, SD = 3 years). For this new group of children, physiological signals were recorded while the Stroop test was administered. Unlike the anagram test used in Experiment 1, this test required no hand movements from the children, who were instructed to limit their movements. The Stroop test has been used to elicit anxiety in previous research (Ozonoff and Jensen, 1999; Christ et al., 2007; Adams and Jarrold, 2009). Although two different tests were used to elicit anxiety in Experiments 1 and 2, this is a methodological strength, as it is important to know if the findings in Experiment 1 were specific to the anagram task, or could be observed in other anxiety-eliciting tasks. The relaxed condition, a low-intensity video remained the same as in Study 1. Means of the physiological signal features of interest are provided in Table 4. There was no significant difference between means of signals from typically developing children and children with ASD in either baseline or anxious conditions. Children self-reported their anxiety state on the State Trait Anxiety Scale—Child version (Spielberger et al., 1970) as previously described. Though this scale was administered to typically-developing children and children with ASD alike, it should be noted that children with ASD may have difficulty recognizing and expressing emotional state (Lang et al., 2010; Hallett et al., 2013). As in Experiment 1, validation of anxiety state required an increase in state score between baseline and anxious conditions. The average difference in normalized state anxiety score observed between relaxed and anxious states was 10.8 points (SE = 3.3) for typically developing children and 8.8 points (SE = 2.7) for children with ASD (Range: For typically-developing children, 44–54 for relaxed and 56–59 for anxious. For children with ASD, 35–56 for relaxed and 51–63 for anxious). This second set of 20 Biomusic samples was combined with the 10 Biomusic samples used in Study 1 to create a total test set of 30 songs. The Experiment 1 Biomusic samples were included in Experiment 2 to explore the potential impact of using two different anxiety-provoking tasks on the results.

**Table 4:** **Means and standard error of physiological features recorded in Experiment 2 from children with ASD (*n* = 5) and typically developing children (*n* = 5) during the relaxed state condition and the anxiety-provoking condition**.

**ASD**		
**Signal feature**	**Relaxed mean**	**Anxious mean**
EDA (μS) ^*^	4.822, SE = 1.627	7.028, SE = 1.577
Skin temperature (°C)	28.424, SE = 1.512	29.251, SE = 1.918
Blood volume inter-pulse interval (s) ^*^	0.744, SE = 0.024	0.686, SE = 0.022
Respiration inter-breath interval (s)	2.211, SE = 0.337	1.616, SE = 0.174
**Typically-developing (Stroop)**		
**Signal feature**	**Relaxed mean**	**Anxious mean**
EDA (μS) ^*^	4.545, SE = 0.924	8.976, SE = 1.902
Skin temperature (°C)	30.208, SE = 2.218	29.467, SE = 1.875
Blood volume pulse inter-pulse interval (s)	0.757, SE = 0.044	0.706, SE = 0.036
Respiration inter-breath interval (s)	2.987, SE = 0.733	1.546, SE = 0.093

* *Indicates features that were significantly different (p < 0.05) between relaxed and anxious state conditions*.

#### 3.1.2. Participants

Twelve of the original 16 participants recruited for Experiment 1 also took part in Experiment 2, which took place approximately 1 month after Experiment 1. The four participants who did not participate in Experiment 2 had completed their terms as students and were not available for follow-up.

#### 3.1.3. Evaluation protocol

Following a short training period (< 5 min) to remind participants of the characteristics of anxious and relaxed Biomusic, participants completed the same task as in Experiment 1, but with all 30 test samples of Biomusic presented in random order.

#### 3.1.4. Data analysis

Data analyses were carried out as in Experiment 1.

## 4. Results and discussion

**Figure 3:**
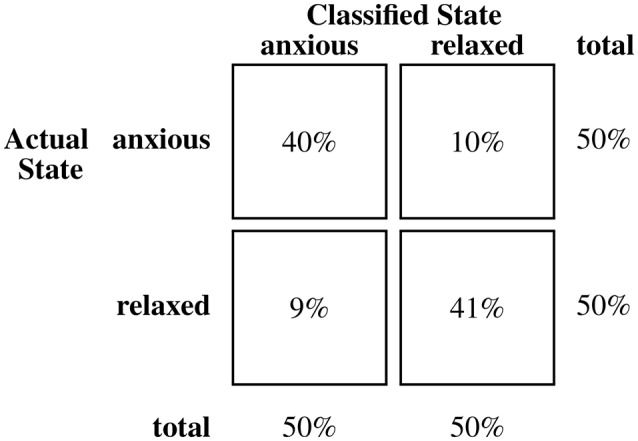
**Confusion matrix for classifications of biomusic from typically developing children (anagram) in Experiment 2**.

**Figure 4:**
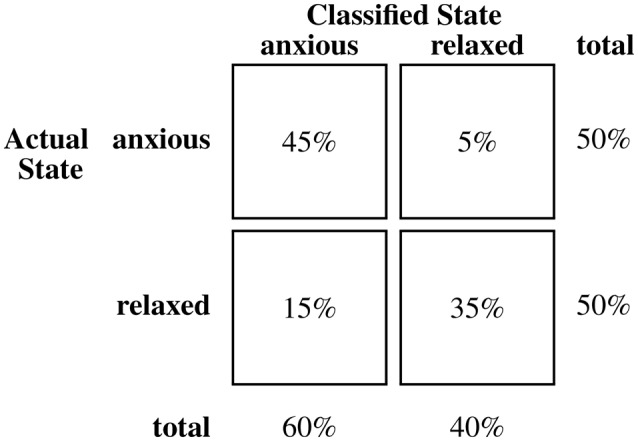
**Confusion matrix for classifications of biomusic from typically developing children (Stroop) in Experiment 2**.

**Figure 5:**
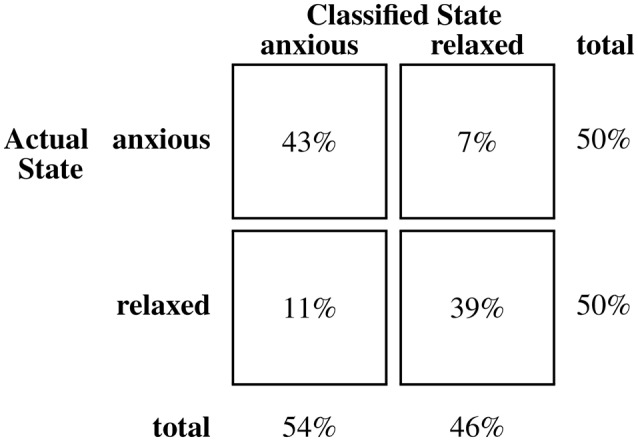
**Confusion matrix for classifications of biomusic from children with ASD in Experiment 2**.

Table 3 presents the means of participants' initial classification accuracy (83.9%, SE = 2.9%), sensitivity (87.1%, SE = 3.4%), and specificity (80.6%, SE = 3.4%). Performance measures for the subset of songs recorded from typically-developing children (**n** = 20) and the subset associated with children with ASD (**n** = 10) are noted. Classification accuracy associated with initial and final impressions and for Biomusic recorded from children with ASD and from typically developing children were comparable (i.e., within 2.5%). On average, participants listened to the Biomusic for 11.3 s (SE = 0.5 s) before completing the classification task. Class confusion matrices (Figures 3–5) demonstrate the errors made by participants during the classification task. False positive errors occured in 10% of classifications while false negative errors occured in 7%.

Participants reported high confidence in their interpretation of Biomusic of typically developing children (median = 4, IQR = 1) and children with ASD (median = 4, IQR = 1). When considering which sounds influenced their classification of the Biomusic sample as anxious- or relaxed-state, participants reported melody (electrodermal activity) as the most influential component for 59.3% of the Biomusic excerpts, followed by drum (heart rate) for 24.8%, chords associated with key change (skin temperature) for 10.9%, and the “whoosh” (respiration rate) for 5.0%. As in Experiment 1, this trend was apparent for both relaxed and anxious state songs.

Thus, Experiment 2 demonstrated that in both typically developing children and children with ASD, anxiety can be conveyed through Biomusic. As in Experiment 1, adults' classifications were made quickly, confidently, and with high performance.

## 5. General discussion

This study aimed to evaluate the performance of the Biomusic interface for identifying distinct patterns of physiological signals associated with anxiety, a clinically significant emotion. This work responds to the need for novel AAC technologies to support communication between caregivers and individuals with profound disabilities. This represents an intermediary, but necessary step toward understanding how/if emotional states can be interpreted from Biomusic.

Classification accuracy, sensitivity, and specificity of the Biomusic interface were high and indicated that participants could reliably classify relaxed or anxious states in typically-developing children and in children with ASD. Participants performed this task with very little (i.e., < 10 min) training and no contextual information. On average, participants listened to the Biomusic for about 11–12 s in both experiments before identifying its affective state. The latency of detection is an important measure to assess the appropriateness of Biomusic for practical applications as it relates to how quickly a caregiver could potentially respond to an anxious child. Because Biomusic itself is not computationally intensive, it has been used in real-time, clinical settings (Blain-Moraes et al., 2013) without introducing computational delay.

Analyses were carried out to identify if certain songs were repeatedly misclassified. Of note, three songs were consistently misclassified above chance. Visual inspection of these excerpts revealed that the physiological signals did not show the expected trends associated with their test condition, suggesting that they were perhaps incorrectly labeled by the child, as opposed to incorrectly classified by the listener. Two of these Biomusic excerpts were associated with typically-developing children (baseline, normalized STAI = 31; baseline, normalized STAI = 49) and one with a child with ASD (baseline, normalized STAI = 56).

Participants reported high confidence in their ability to classify emotional states based upon the auditory information presented through Biomusic. In both experiments, melody (electrodermal activity) was reported as the most influential component in the classification task. This was not entirely surprising as the mapping of skin conductance to melody (one of the most prominent and easily distinguishable musical components) was a design decision made to sonically reflect the salience of EDA patterns in physiological manifestations of anxiety as suggested in previous studies (Kreibig, 2010; Kushki et al., 2013). It is notable that we deliberately designed physiological-musical mappings that attempted to preserve the natural qualities and connotations of the biosignal in question (e.g., expiration length was matched to an embellishment that resembled the sound of an exhalation). Such considerations may improve the ability of users to remember and interpret the significance of musical changes. However, future research is required to determine if other physiological-musical mappings could further improve accuracy, particularly for individuals with atypical electrodermal activity responses. Interestingly, the electrodermal activity of children with ASD has been reported to respond atypically to anxious situations (Kushki et al., 2013). However, participants in Experiment 2 were able to classify signals of typically developing children and children with ASD with similar high performance.

Of note, the initial classification sensitivity of the Biomusic (89.7% in Experiment 1, 84.9% in Experiment 2) compares well with that of the established, observational scale, the modified Yale Preoperative Anxiety Scale (85%), which has been previously validated against the gold-standard State Trait Anxiety Inventory for Children (Kain et al., 1997). Accuracy is also similar to that achieved via algorithmic pattern analysis of physiological signals, though direct comparison is often difficult due to methodological differences between studies (Wen et al., 2014). For example, Rani et al. (2006) evaluated K Nearest Neighbor, Regression Tree, Bayesian Network, and Support Vector Machine algorithms for the detection of anxiety in healthy adults, elicited using an anagram test. Inputs for these classifiers were chosen by hand on a case-by-case basis for each participant, due to physiological response variability across participants. Accuracy of these classifiers ranged between 80.38 and 88.86further research with larger and more heterogeneous samples is warranted, our Biomusic system appeared to be comparable for anxiety detection in typically-developing participants and those with ASD. This potential for generalization is essential for the intended use scenario where there is a high potential for condition- and person-specific variability, but it may be impossible to train person-specific algorithms given the lack of self-report.

### 5.1. Study limitations and future work

Note that generalizations of this study should be made with caution. While the target user for Biomusic is a child with profound disabilities, having possibly typical or atypical physiological signals and no ability to self-report emotional state, this presents a paradox to system validation: the children most in need of the Biomusic interface are also the population for which Biomusic development/evaluation is most challenging. As such, it is important to first establish the performance of the Biomusic interface where self-report is possible in order to maximize our understanding of the technology and potential success when translating to clinical populations with more severe communication challenges. Although gold standard measures were used to define anxious and relaxed states through self-reports, it is near impossible to ascertain these emotions with absolute certainty, even in the typically-developing group. While in the majority of cases, visual analysis of the physiological signals also supported the assigned classification labels, there were three notable exceptions. These songs were all associated with poor classification accuracies, which may indicate that the overall detection accuracies reported are quite conservative.

The two training samples were not used in the testing phase of either experiment. However, separate data segments from the same child were used to generate two distinct Biomusic samples that were included in the test set. Plots of the training and testing segments of this child's physiological signals are included in the Supplementary Materials. Three researchers listened to the Biomusic prior to conducting these experiments and were unable to discern a child-specific “signature” to the sonification that could contribute to a learning effect. If such a signature is present, it is unlikely that it could have been discerned with a single 80-s sample of an anxious and relaxed state as presented in participant orientation. The classification accuracy of this child's test samples was 82%. For comparison, the average classification accuracy of children's test samples was 82% in the typically developing group that completed anagrams. The average classification accuracy of all children's test samples was 81%.

In a follow-up questionnaire, we asked the 16 adult participants the question, “Are the songs pleasant to listen to?” 15 participants responded “Yes,” indicating that Biomusic is not only informative, but aesthetically appealing to the listener. Further research will be needed to probe the aesthetics and usability of Biomusic in a care context.

Given the promising results of this study, future research is warranted with larger and more diverse samples of children and listeners (e.g., parents, nurses, therapists, physicians) in a real-time context. It would be interesting to explore if Biomusic has the specificity required to distinguish high intensity/negative valence states (e.g., anxiety) from high intensity/positive valence states (e.g., excitement), or if valence is more effectively identified through contextual cues. Our experiment was not designed to explore multiple emotional states or to evaluate how well transitions between emotional states can be detected. The complexity of affective responses (e.g., prevalence of mixed emotions and the fact that transitions between emotions in practice can rarely be discretely defined as the intensity and valence of emotions are often considered to lie on a spectrum) makes these experiments extremely difficult to design and carry out, particularly in populations where self-report is a challenge. The complexity and continuous nature of affective responses is in fact one of the motivators underlying the development of our Biomusic interface which can present multiple physiological signals continuously as a supplement to overt affective responses and contextual information. This study was designed to investigate the usability and validity of the interface in a well-controlled environment prior to deployment in more contextually-rich and clinically relevant environments.

Our anxiety-provoking test conditions generated Biomusic samples that were distinguishable from relaxed-condition samples, even though elicited anxiety was likely moderate. For reference, the children in our samples had an average normalized state anxiety score of 55.9 points during the anxiety eliciting tasks. For children in another study who were told to imagine a school test-taking scenario (*n* = 913), the average normalized state anxiety score on the STAI was 41.8 points for males and 43.8 points for females (Spielberger et al., 1970). For children pre-surgery (*n* = 39), average normalized state anxiety scores of 69.89 and 71.90 points in females have been reported (Alirezaei et al., 2008). In comparing these different contexts, we hypothesize that the tasks used in this study were only able to elicit moderate increases in anxiety. Future work, likely in real-world contexts, are needed to explore the utility of the Biomusic interface across a wider range of intensities and emotions.

Further, it would also be important to evaluate the performance of the system during a real-time caregiving activity where contextual and behavioral cues are also integrated. Qualitative studies would be well-suited to probe these lines of investigation. Future studies could also assess whether sensitivity, specificity, and accuracy of detections remain high when individuals must engage in other tasks as is typical in real-life care-giving settings and if Biomusic is an acceptable interface for long-term use. Biomusic may also show promise as a tool for emotional self-regulation, particularly for children who struggle to identify and express their emotional state. This is an area worth further exploration.

## Author contributions

SC, EH, and EB developed the study protocols. EA and AK contributed to study design for Experiment 2. SC, EH, and EB wrote the manuscript. SC and EH contributed equally to this paper.

## Funding

The authors report no declarations of interest. This work was supported in part by a Discovery Grant from Natural Sciences and Engineering Council of Canada (#371828-11); the Ontario Ministry of Training, Colleges and Universities through an award to EH; the Holland Bloorview Kids Rehabilitation Hospital Foundation; and the Ward Family summer student research program through an award to SC.

### Conflict of interest statement

The authors declare that the research was conducted in the absence of any commercial or financial relationships that could be construed as a potential conflict of interest.
