# Supplementary material for: Biomusic: An Auditory Interface for Detecting Physiological Indicators of Anxiety in Children
Source: Front Neurosci. 2016 Aug 30;10:401. doi: 10.3389/fnins.2016.00401 (PMC5003931; doi:10.3389/fnins.2016.00401)
Supplement: Supplementary file 3 [file Image1.PDF]

Supplementary Material: Biomusic: An auditory interface for detecting physiological indicators of anxiety in children

Stephanie Cheung [1,2,†,*], **Elizabeth Han** [1,2,†], **Azadeh Kushki** [1,2], **Evdokia Anagnostou** [2,3], **Elaine Biddiss** [1,2]

*Correspondence:
Stephanie Cheung
scheung@hollandbloorview.ca

† Contributed equally to this paper.

1 BIOMUSIC SAMPLES

Samples of Biomusic are provided. AUDIO1.wav is an example of anxious Biomusic, generated from physiogical signals of a typically-developing child in Experiment 1. AUDIO2.wav is an example of relaxed Biomusic, generated from the same child.

2 PHYSIOLOGICAL SIGNALS

Plots of physiological signal segments are provided for the typically developing child whose signals were used to generate both training samples and testing samples of biomusic.

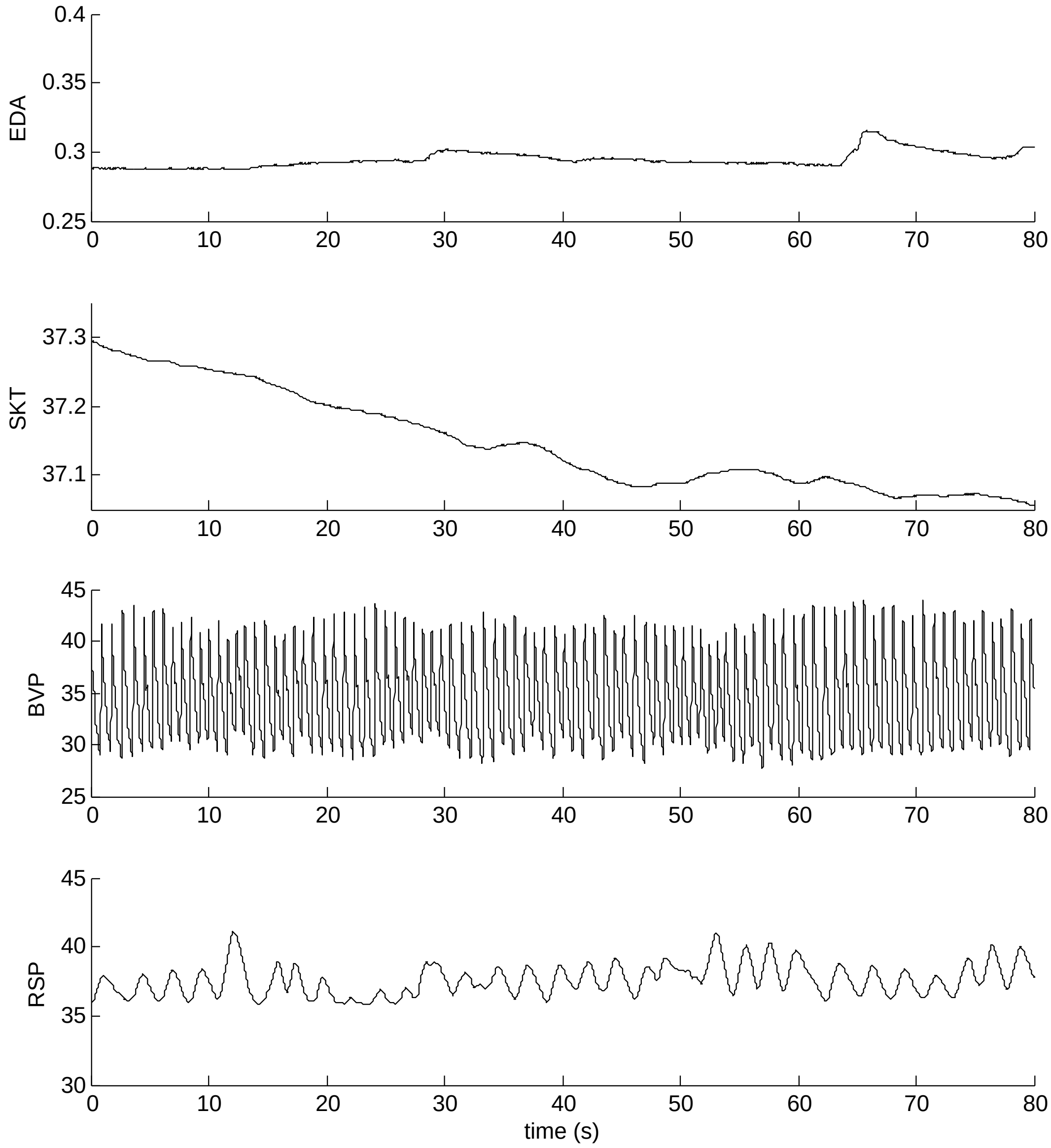

Figure S1. Raw relaxed physiological recordings (electrodermal activity EDA; skin temperature SKT; standardized blood volume pulse BVP; standardized respiration RSP) from a typically-developing child, before conversion to a biomusic training sample.

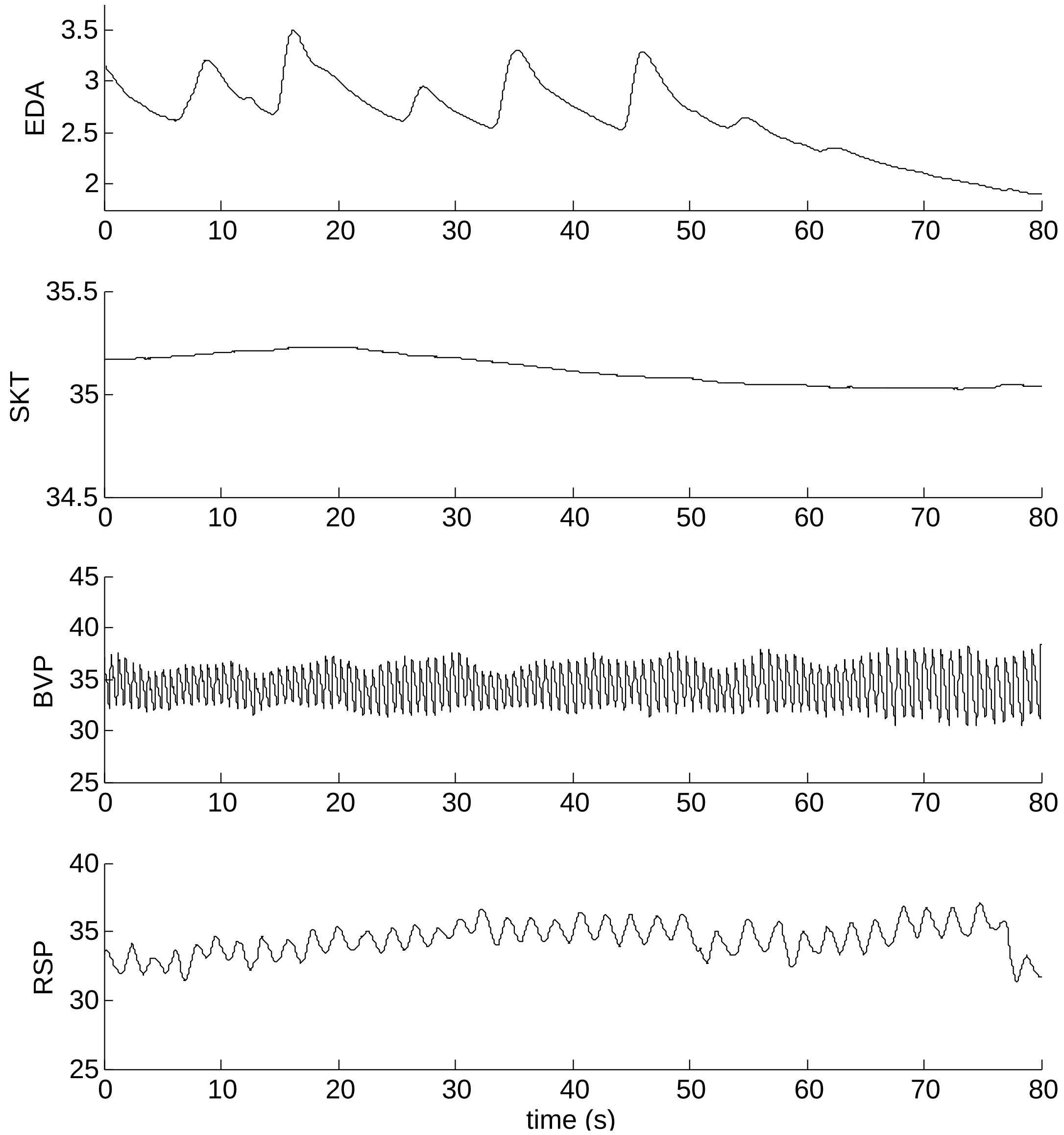

Figure S2. Raw anxious physiological recordings (electrodermal activity EDA; skin temperature SKT; standardized blood volume pulse BVP; standardized respiration RSP) from a typically-developing child, before conversion to a biomusic training sample.

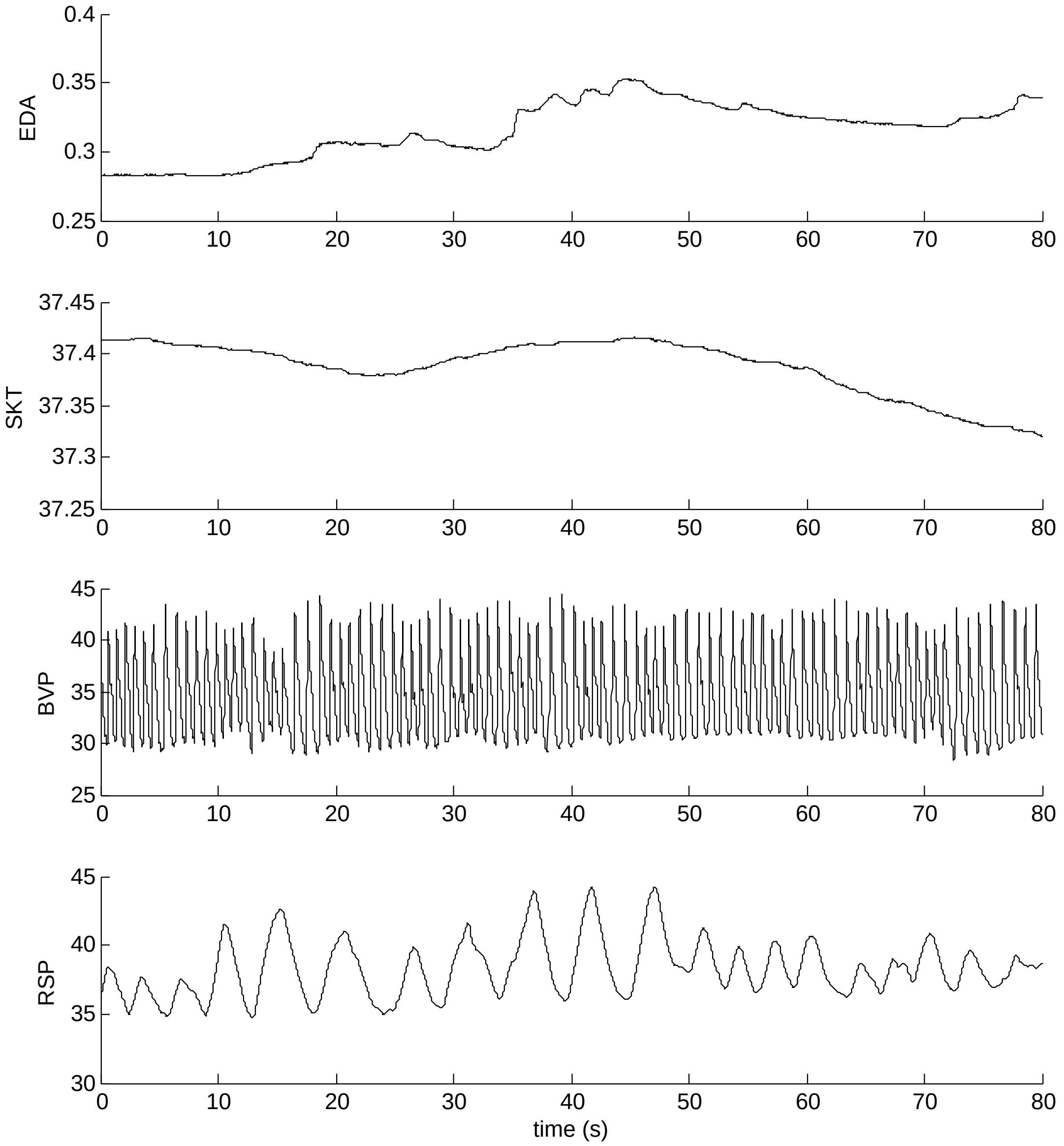

Figure S3. Raw relaxed physiological recordings (electrodermal activity EDA; skin temperature SKT; standardized blood volume pulse BVP; standardized respiration RSP) from a typically-developing child, before conversion to a biomusic test sample.

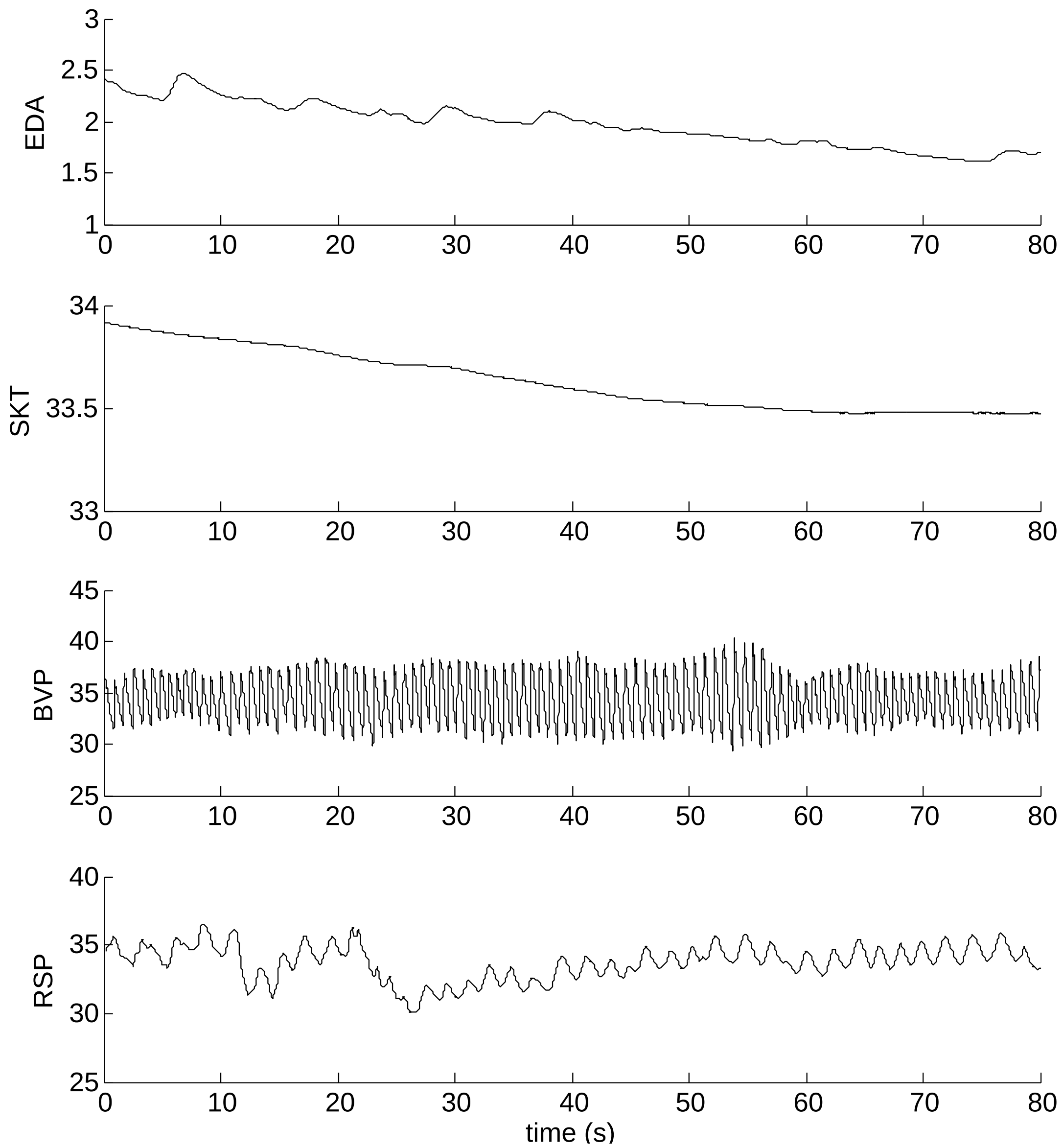

Figure S4. Raw anxious physiological recordings (electrodermal activity EDA; skin temperature SKT; standardized blood volume pulse BVP; standardized respiration RSP) from a typically-developing child, before conversion to a biomusic test sample.
